# Gesture-Based Human Machine Interaction Using RCNNs in Limited Computation Power Devices

**DOI:** 10.3390/s21248202

**Published:** 2021-12-08

**Authors:** Alberto Tellaeche Iglesias, Ignacio Fidalgo Astorquia, Juan Ignacio Vázquez Gómez, Surajit Saikia

**Affiliations:** 1 Computer Science, Electronics and Communication Technologies Department, University of Deusto, Avenida de las Universidades 24, 48007 Bilbao, Spain; ivazquez@deusto.es; 2 DeustoTech-Deusto Institute of Technology, University of Deusto, Avenida de las Universidades 24, 48007 Bilbao, Spain; ignacio.fidalgo@deusto.es (I.F.A.); surajit.saikia@deusto.es (S.S.)

**Keywords:** real time, deep learning, gesture detection, embedded systems

## Abstract

The use of gestures is one of the main forms of human machine interaction (HMI) in many fields, from advanced robotics industrial setups, to multimedia devices at home. Almost every gesture detection system uses computer vision as the fundamental technology, with the already well-known problems of image processing: changes in lighting conditions, partial occlusions, variations in color, among others. To solve all these potential issues, deep learning techniques have been proven to be very effective. This research proposes a hand gesture recognition system based on convolutional neural networks and color images that is robust against environmental variations, has a real time performance in embedded systems, and solves the principal problems presented in the previous paragraph. A new CNN network has been specifically designed with a small architecture in terms of number of layers and total number of neurons to be used in computationally limited devices. The obtained results achieve a percentage of success of 96.92% on average, a better score than those obtained by previous algorithms discussed in the state of the art.

## 1. Introduction

Gestures are key elements in the fields of interaction, understanding, and communication with machines. In certain situations, where other kinds of communication fail, such as speech recognition with environmental noise, gesture processing approaches have demonstrated to be a valid strategy, also offering the benefit that they do not need the use of additional elements or components to acquire extra data.

Gestures can be defined as natural movements made by humans, presenting many variations when done, either when the same person is performing the same gesture for several times, or when different people are doing it. Also, in the case of using images, variable environmental conditions add more challenges to the gesture detection process.

A gesture recognition system must be also flexible enough to recognize new gestures and must support the training of new ones, and because of these needed features, complex processes are needed for gesture recognition like motion modelling or pattern recognition. Some efforts on gesture recognitions started in 1993, where some techniques were adapted from other fields like speech or handwriting recognition. In this way, Darrell and Pentland [1] adapted Dynamic Time Wrapping (DTW) to recognize dynamic gestures. After that, Starner et al. [2] proposed to classify orientation, resultant shape and trajectory information of the gesture, using Hidden Markov Models (HMM).

In specific gesture-detection applications, different wearable sensors have been developed, such as gyroscopes or accelerometers. In [3], the authors use magnetic and inertial sensors placed in a glove to capture the movements of the hand and arm. In [4], a stretch-sensing soft glove is presented to perform an interactive hand-position capture. This method presents high accuracy without any other external optical system. They also demonstrate the way to make a calibrated low-cost version, using common components available in most of the manufacturing labs. On the other hand, the use of image-processing algorithms and point clouds for gesture and posture recognition is becoming possible by using 3D cameras, sensors providing point clouds or more common color images. In [5], the segmented skeleton joints are used in combination with a precise segmentation of depth images to extract gesture information. On the other hand, in [6], the authors use segmentation of color in 2D images to detect hands, head or tags. Ge et al. [7,8] introduce a new method for a real-time pose estimation using 3 dimensional Convolutional Neural Networks (CNNs). This approach takes a 3D volumetric representation of the hand, using a depth image as input, and then it extracts three-dimensional features from the volumetric input, capturing the 3D spatial structure of the hand, and in the same way obtaining the 3D pose of the hand using a single pass. Finally, and in the same way, the Google Media Pipe framework uses deep learning to track and detect gestures performed with both hands [9].

There are some methods that use a random forest algorithm [10,11] or autoencoders [12] for the estimation of the structure of the hands. More recently, there are some works that adopt a more computationally efficient methods based on these hierarchical structures [13].

In recent years, research works try to detect gestures by detecting hand and head of persons. In [14], head and hands of the users are tracked using the 3-dimensional space. In the same way, in [15], a color segmentation using 2D images is used to detect user’s hand. In [16] the detection of the hands is performed by using both approaches.

Another field of interest in this research area is sign language recognition, which is a sub-field of communicative gestures. Since this type of language is highly structural, it is frequently used in computer vision algorithms. As an example, Cheok et al. [17] proposed an alignment network to detect specific hand gestures with iterative optimization for weakly supervised continuous sign language recognition. This solution presented two different approaches working together: a 3D convolutional residual network for feature learning and an encoder-decoder network used for sequence modelling. Also, works presented in [18,19], present complete reviews of different algorithms and specific approaches intended for gesture detection applications.

As exposed above in this introduction, deep learning algorithms represent the latest big advancement in the artificial intelligence and pattern recognition fields. These techniques are based on the construction of highly complex neural network architectures, with the final objective of replicating the inference and abstraction capacities of the human beings in varied tasks such as object recognition, scene interpretation or text and speech recognition and generation.

The first Convolutional Neural Network, created for image processing tasks and named AlexNet, was presented by the research group leaded by Geoffrey E. Hinton at Toronto University. With it, they won the ImageNet Large Scale Visual Recognition Challenge [20].

In this sense, the CNNs have become one of the best solutions to solve intricate image processing problems among a great variety of applications fields [21]. Nowadays, it is easy to find many different architectures, to perform image processing tasks, such as GoogleLeNet [22] developed by Google, VGG [23], ResNet [24] engineered by Microsoft, or RCNNs (Region based CNNs).

This last one is the most common approach based on CNN to detect objects in images. More concretely, the most common approaches for object detection applications can be grouped as region proposal-based methods, such as RCNN and its derived algorithms, SPP-net or FPN, or regression/classification methods as YOLO, Multibox, G-CNN, AttentionNet, and so on [25].

The region proposal methods include multiple internal steps, such as proposal generation, feature extraction and classification and bounding box regression operations. The RCNN based models are examples of this method [26]. In this specific case, it has three main evolutions to obtain optimum results in object detection: RCNN, Fast RCNN, and Faster RCNN.

**Figure 1:**
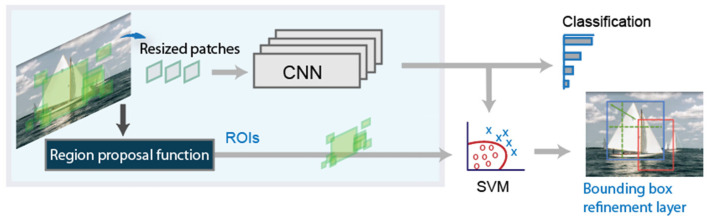
Basic schema of the RCNN detector.

The original RCNN approach first generates region proposals using image processing algorithms such as Edge Boxes [27]. In the paper defining the RCNN architecture, the authors use tentatively 2000 regions for each image. Each of these generated regions are cropped and resized to be classified by a CNN. In the last step, the bounding boxes proposed are refined by a support vector machine (SVM) previously trained with the CNN features. A schema of this system can be observed in Figure 1.

**Figure 2:**
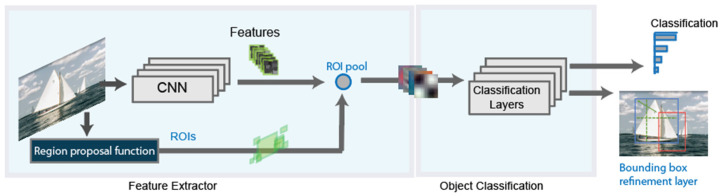
Basic schema of the Fast RCNN detector.

A refinement on the RCNN detector, is the Fast-RCNN detector [28]. Figure 2 shows the new detector architecture. The principal difference with the basic architecture is that, after the region proposal function, instead of cropping and resizing region proposals, Fast-RCNN detector analyzes the complete image. The Fast-RCNN algorithm pools the features of the proposed regions using a CNN. This makes Fast-RCNN more efficient, because there are not recurrent computations in overlapping regions.

**Figure 3:**
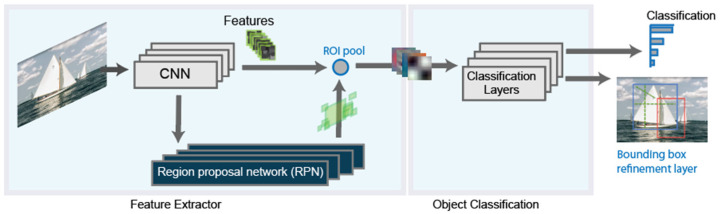
Final schema for the Faster RCNN detector.

The most recent evolution of the RCNN algorithm is the Faster-RCNN [29], shown in Figure 3. In this case, a region proposal network is used in a direct way. With this, the network is faster, and it is better adapted to the training data, offering faster, and better results.

We selected the different variations of the RCNN object detection algorithm in this work because their structure is based on an internal CNN network that can be created taking into account the computation capability available, making this approach fully adaptable to limited hardware.

Section 2 exposes the precise use case presented in this article. Section 3 explains the dataset used for the training stage of the proposed solution. Section 4 explains the proposed approach in this paper in terms of network architecture. Section 5 presents the training procedure of the different RCNN alternatives, and in Section 6, the results obtained after the evaluation tests are presented. Finally, the last section, Section 7, summarizes the conclusions obtained from this research, highlighting the strong points of the proposed method.

### Main Motivation and Contributions

The main motivation of this work is to design an effective CNN architecture that can be deployed and used in computationally limited devices, offering real-time gesture detection capabilities in embedded devices with the previously cited limitation.

Following this motivation, this article presents a research work to analyze the performance of the different RCNN approaches for the detection of hand gestures in real time, with the final objective of obtaining a lightweight system that fits not only in desktop PC, but also can be run in IoT (Internet of Things) devices like, Google Coral or Nvidia Jetson platforms, providing a real-time response.

After the testing of different previously existing networks, the designed CNN presents a very small architecture both in terms of number of layers and total number of neurons, offering at the same time real time capabilities and a percentage of correctly detected gestures of almost 97%, overcoming results in state of the art. This is the main contribution of this work.

## 2. Problem Statement

One of the biggest problems that Deep Learning techniques present in general, and CNNs in particular, is the high computational requirements. Several researchers pointed out this problem, like [30] or [31].

When we face a problem that requires a short time response, like in this case, small CNNs can be good candidate models to solve in the RCNN detectors when we try to validate the system’s performance. Before analyzing several different networks present in state of the art, small CNNs provide also smaller footprint, particularly if we compare them with other architectures (e.g., Squeezenet [32], GoogleNet [22], and Mobilenetv2 [33]). In Table 1, we present some of the principal features that these networks have. In the depth column present in the table, we defined the largest number of layers (fully connected or convolutional) on the path from the first to the last layer.

The RCNN detectors will detect the different user hand positions and will return as output the class of the gesture detected with the best accuracy.

Finally, as a summary, we can highlight three relevant features needed when developing a gesture detection system. These are the following:

- Capability to define new gestures quickly. This feature provides flexibility to the system.
- Flexibility to identify the same gesture made by different people, with a different orientation, skin color, hand position, and other aspects of the environment, like the light of the place or different distances to the performed gestures.
- Due to the real-time constraints in several scenarios, the system has to provide a fast response.

Despite having enough computation power to deploy systems based on small-sized CNNs, the biggest issue with these embedded systems is related to the software. These devices only support a limited version of the traditional Deep Learning frameworks, like Tensorflow Lite, or new frameworks developed to maximize the performance of these devices, like OpenVino. These reduced versions do not support all the CNN layers that are available in the scientific literature.

According to these limitations, the developed architecture must fulfil different conditions to be compatible with these devices:

- Use standard CNN layers to avoid the limitations of the lite version of the frameworks. Due to the resources constraints, it is essential also to have a reduced size.
- Improve the response time, avoiding hardware constraints of these platforms.
- Compatibility with transfer learning techniques, with pre-trained models, to minimize the training step.

## 3. Specific Dataset for Hand-Gesture Detection

There are a lot of gesture datasets already at researchers’ disposal, but, many of them, due to their research purposes, do not represent the real-world conditions correctly, especially in tasks that involve human-machine interaction. Some of these datasets are, for example, the dataset created by the University of Padova using Kinect and Leap Motion devices [34], The Hand-Gesture Detection Dataset, created by the Video Processing and Understanding Lab [35], or the Hand Gesture Database [36]. In them, the authors have already preprocessed the images, and the hands are segmented, performing different gestures. Because of this, researchers do not need to locate hands in the overall scene. Hence, these datasets do not have a feasible application in systems that want to detect real-time gestures when working in an environment that has to be constantly under inspection.

The main application presented in this article is in the area known as “human-machine interaction” (HMI). In this type of application, the number of gestures that these systems need to identify is usually limited. For example, manual control of a robot operation needs four or five principal commands, such as “stop”, “resume”, “start”, or “reset”. In other application examples, the situation is just similar: in a television control based on gestures, a few of them are needed, “switch on/off”, “volume up/down”, “program up/down”. Using a great number of classes (i.e., gestures) makes the understanding of these systems a hard task, and their practical usability decreases.

In this work, we present a dataset that consists of four different commands done by gestures, defined assuming the typical interaction carried out between a collaborative robot and a worker in an industrial setup [37]. The four different gestures: ‘Agree’, ‘Halt’, ‘Ok’ and ‘Run’. The position of fingers and the hand represent each gesture. Table 2 presents the hand positions for each gesture.

**Figure 4:**
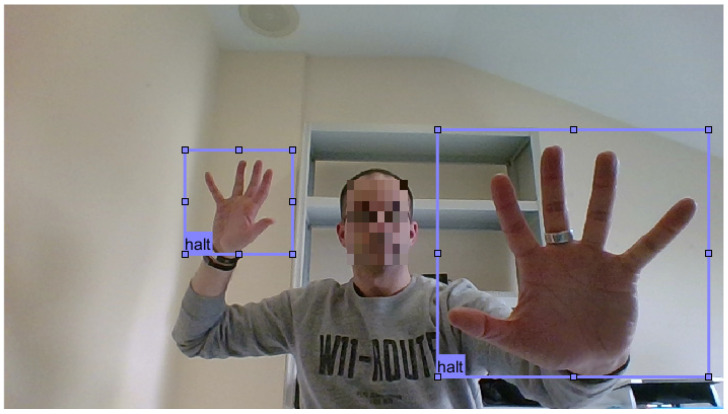
Image for the Halt gesture.

For each gesture, we recorded and labelled 100 different images. In each image, we use both hands to perform the same gesture, so we labelled a final amount of 200 gestures for each class, with a total of 800 gestures in our training dataset. Using both hands in each image allows the learning generalization and adds more samples to the dataset. We also labelled the gestures at different distances from the camera, adding more variability. Figure 4 shows an example of an image that has been labelled for the Halt gesture.

The dataset is created with both the image name and the coordinates of each labelled gesture as bounding boxes, following this schema: (x, y, w, h), where x and y represent the coordinates, using the upper left corner as a starting point, w is the width and h the height of the bounding box. All 400 images (100 images for each of the 4 classes) in the dataset have the same resolution: 1280 × 720 pix.

One problem that must be considered is the overfitting problem that can happen when a small number of classes and a CNN with a small number of trainable parameters are used. This situation, which is an important problem in generic gesture detection applications, can be assumed in HMI applications. Overfitting itself presents an over adjustment to the dataset. In the HMI application configured with a specific dataset created by the future users of the system, this over adjustment cannot be taken as a drawback, but even as an advantage in scenarios where the operation security is critical.

## 4. Proposed Network Architecture

We propose a new optimized CNN architecture, evolving the Darknet reference model presented in [38]. This Darknet architecture’s main feature is the speed on the detection stage while having a relatively contained number of trainable neurons and a simple network architecture. Despite the limitations exposed in Section 2, this model suits our application. Using this model allows us to minimize the training process, using a transfer learning approach with a pre-trained model of the network [39].

**Figure 5:**
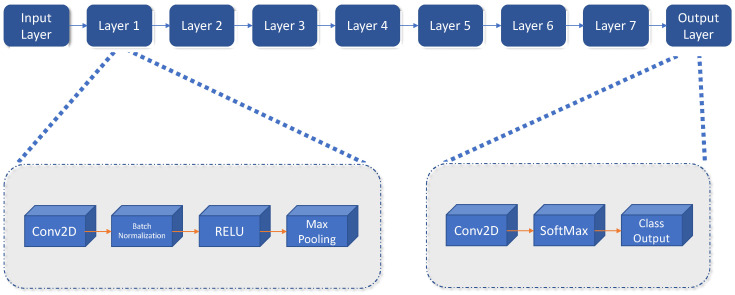
Schema of the Darknet network model.

The Darknet network is composed of 32 layers. Figure 5 shows the schema we have used. On the other hand, Table 3 shows the different filters located in the eight convolutional layers.

**Figure 6:**
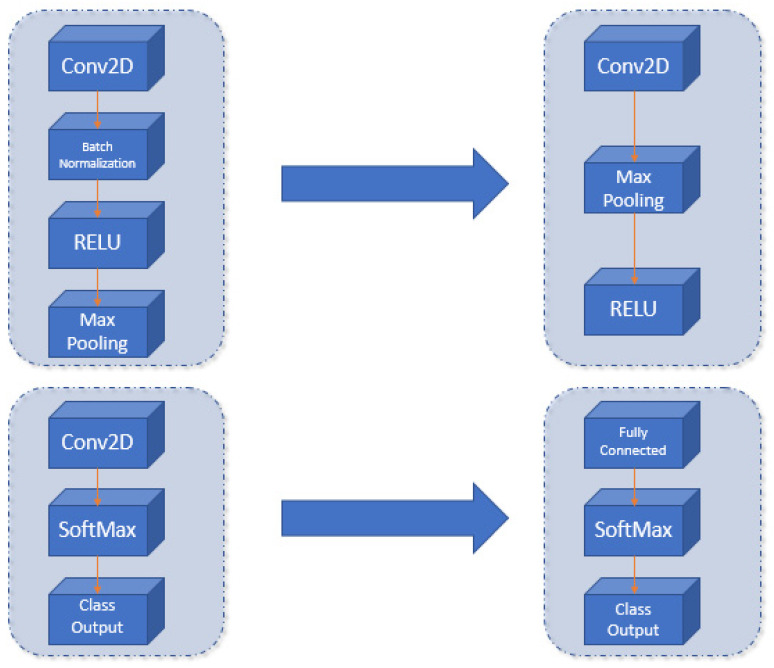
Proposed changes in the Darknet architecture (**left**) to create the new CNN (**right**).

The proposed CNN in this work wants to simplify the Darknet model and minimize the training step, using a transfer learning approach, maintaining the Darknet convolutional layers’ trained weights. The carried-out changes are the following:

- Simplification of intermediate convolutional layers (shown in Figure 5). The embedded platforms framework limitations justify this simplification of layers. Thanks to this adaption, it makes the network smaller and more portable to many different systems.
- Modify the output layer of the network. We used a fully connected layer of 5 outputs (number of gestures to detect and one extra layer, needed to make the model more usable by the RCNN detectors) (shown in Figure 6).

Due to these modifications, the proposed network has only 25 layers instead of the 32 layers in the original network.

Table 4 shows the comparison between the proposed solution and state of the art in the small CNNs area, explicitly created to be used in embedded devices.

The proposed network in this work has a similar number of parameters if compared with other small CNNs, but it has a significant lower number of layers. Parallelizing the calculations needed for each layer, the proposed network is much more efficient in computation time, as we stated in Section 6. SqueezeNet has almost three times more sequential layers than our proposed network (68 layers versus 24), and MobileNetv2 has even more, a total amount of 155 sequential layers, 6.45 times more than our proposed approach. Despite these two well known architectures are optimized in the number of trainable parameters, they present a more sequential structure, offering less possibilities for parallelization and hence, requiring more computation time than our optimized network.

## 5. Training of the Faster RCNN Object Detector

The training parameters have been set up following the criteria revised in related works using similar architectures. They have been later validated taking into account the results obtained by the different network models that have been used for testing. Next section exposes in detail internal network parameter settings used in the algorithm training procedure.

### Setting Up of Modified Darknet Network Parameters

The algorithm used to train the modified Darknet CNN to be used in the Faster RCNN detector has been the stochastic gradient descent with momentum (*sgdm*) optimizer. The original version of this algorithm was firstly presented in [40].

This iterative mathematical procedure updates network parameters, weights and biases, minimizing the loss function moving towards the negative gradient of this function value as presented in Equation (1):

(1) $$\theta_{i+1}=\theta_{i}-\alpha \nabla E\left(\theta_{i}\right)$$

where *i* is the iteration number, *α* > 0 is the learning rate, *θ* is the parameter vector and *E*(*θ*) is the loss function.

The first original version of this algorithm presented the problem of oscillations in the followed path to reach the optimum.

Adding a momentum term to the original sgd algorithm can minimize this problem [41], leaving the mathematical expression for this optimizer as follows:

(2) $$\theta_{i+1}=\theta_{i}-\alpha \nabla E\left(\theta_{i}\right)+\gamma \left(\theta_{i}-\theta_{i-1}\right)$$

where *γ* is the momentum value that determines the contribution of the previous gradient step in the current iteration.

To carry out our research, we selected the following values for each parameter: *α* = 0.001, *γ* = 0.9. A learning rate with a small value allows the correct convergence of the training stage in a more robust way.

The loss function used is cross entropy or multinomial logistic loss, shown in Equation (3).

(3) $$H\left(P_{n},Q_{n}\right)=-\sum_{i}^{}P_{n}\left(i\right)logQ_{n}\left(i\right)$$

where $P_{n}$ is the multiclass predicted vector for the sample n and *Qn* is the desired output. i is the number of different classes in the problem under study.

In this case, the *sgdm* algorithm is applied using a small-batch strategy in the training stage, to evaluate the gradient value. Because of memory limitations, the batch size has been established to 8, and the number of epochs to 40 in Faster-RCNN approach.

Finally, the number of regions proposed in the RCNN detectors´ algorithms has been set up to 1000, half its original value of 2000. This smaller value does not harm the effectiveness of the detection, and presents a great advantage in memory and computation time optimization.

**Figure 7:**
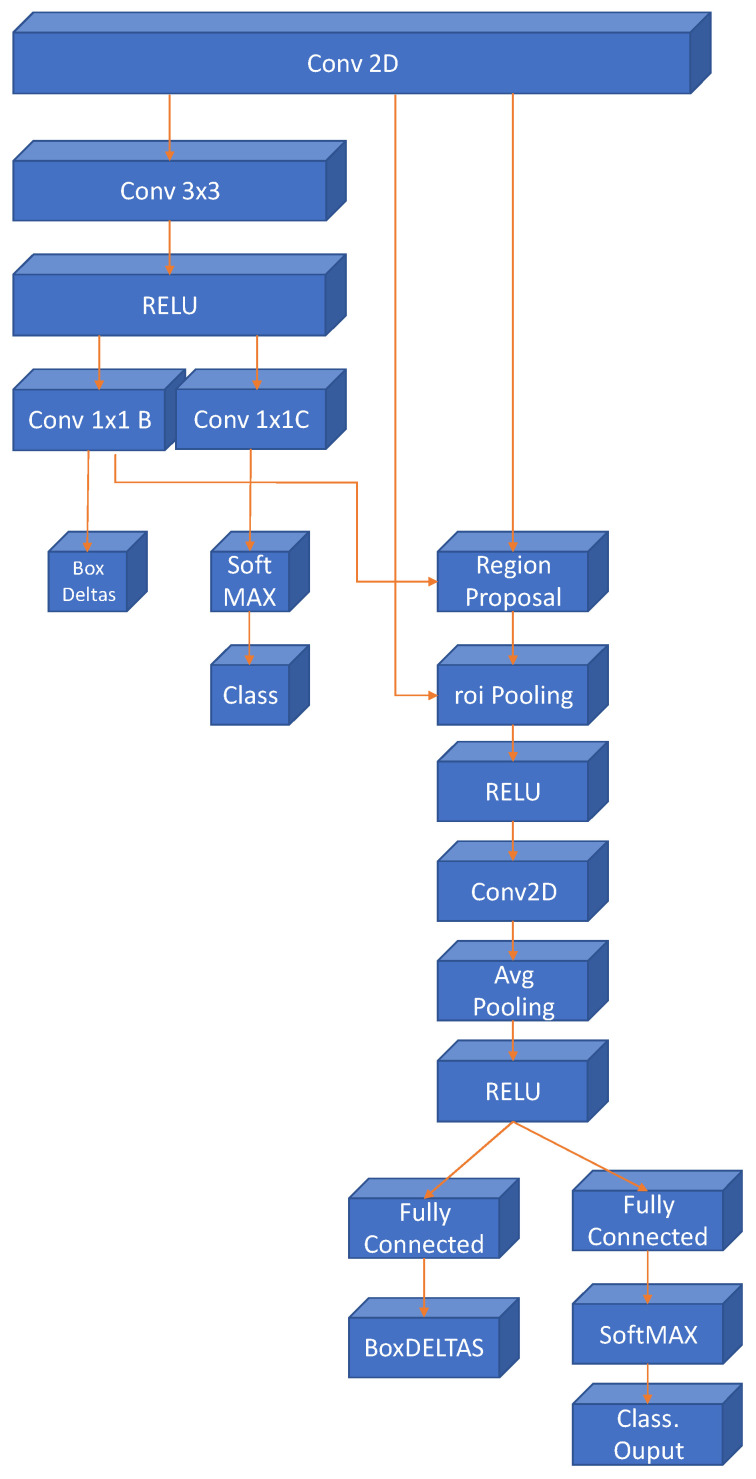
Final layers of the gesture detector using the proposed CNN in this work in a Faster RCNN architecture.

Figure 7 shows the final network architecture obtained for the Faster RCNN classifier. The convolutional layer at the top of the image corresponds to the 6th convolutional layer of the original network, the latest layers have been created by applying the Faster RCNN architecture, using as its core the proposed network in this study. The final network provides two outputs the coordinate boxes where the gestures are located and the gesture class.

## 6. Discussion of Results

Different gesture recognition approaches have been trained using a Nvidia GeForce 1080 Ti graphics card to take advantage of the parallel computation, in a PC with Ubuntu Linux 20.04 LTS and MATLAB R2020b. Specific tools provided by MATLAB for the development of Deep Learning applications and for parallel computation have been applied for network design and testing.

### 6.1. Proposed CNN Architecture Performance Assessment

The constructed dataset has been divided in two smaller parts: training and testing. The 800 gestures available have been randomly shuffled, using 120 of them (15% of the dataset) for testing, and the remaining 680 for training.

Finally, transfer learning techniques have been applied for pretrained models and proposed network in this work. Comparison with the original Darknet model has not been taken into account for comparison metrics because the model created for this work is precisely an optimization of this network, and subsequently it is assumed a better performance of the newly created architecture.

Results tables below present the results for the different gesture detection approaches and different networks used. The numerical results presented are:

- Percentage of correctly classified instances: The gesture is correctly detected and located in the image. Failure in detecting a gesture, or detection of a different one is considered the same error. This metric is evaluated using the Correct Classification Percentage (*CCP*) formula:
  (4) $$CCP=\frac{TP+TN}{TP+FP+TN+FN}$$
  where *TP* stands for *True Positive*, a gesture correctly detected. *TN* means *True Negative*, no gesture is detected if there is an absence of gestures. *FP* is a *False Positive*, a gesture is detected incorrectly, and finally, *FN*, *False Negative*, is a gesture performed not detected.
- Accuracy calculated in gestures detected correctly: Mean reliance obtained in correctly detected gestures.
- Time for detection of gestures, per frame. Time calculated as the mean detection time for the 120 testing gestures.

Attending to the results presented in Table 5, Table 6 and Table 7, the proposed CNN architecture surpasses results of state of the art networks in gesture detection and execution time in RCNN and Fast RCNN architectures.

The Faster RCNN detector, is also better than any other tested network in execution time, and its classification results indicate the same performance that the reference networks have.

In the case of use of a Faster RCNN detector with the proposed CNN, obtained detection time is 0.14 s, which provides a capacity to analyze 7 images per second. This execution time combined with a 98.33% in detection accuracy makes this detector a very valuable option for real-time gesture recognition.

### 6.2. Proposed CNN Architecture Performance Evaluation

Partial results presented in Section 6.1 have validated the proposed detector based on the Darknet model, obtaining detection results equivalent to the ones obtained by the selected reference networks, being smaller and faster at the same time.

With the presented architecture validated, this section shows validation results more precisely, applying a 10-fold cross validation strategy with the objective of obtaining more precise measurements of correct classification instances, accuracy in detection and detection time.

The dataset, as explained in Section 3, is composed by 400 images with a total number of 800 gestures. 10-fold cross validation approach fits then with training groups of 720 gestures and a validation subset of 80 gestures, for each of the 10 iterations.

Table 8 presents the results obtained for each fold and the average final results of the network performance, using the Faster RCNN detector.

As it can be inferred from these presented results, the proposed architecture offers a good balance in the CCP metric, accuracy in detection and speed, and can be considered an alternative for HMI systems in real time.

## 7. Conclusions and Future Work

CNNs have demonstrated to be a very powerful option to solve complex image processing problems that present high variability. Partial occlusions, perspective problems, variability in sizes of the same object due to distance, changes in color and lighting, etc., are examples of these type of situations.

These networks have had an outstanding performance in many proposed challenges and competitions, such as ILSVRC [39], the challenges presented in the Kaggle platform [42] or The Low-Power Image Recognition Challenge [43].

On the other hand, the principal drawback of this technology is that high computation capacities are needed for the majority of the problems that use CNN. This leaves systems like embedded systems of industrial equipment out of the potential areas that can take advantage of modern neural computation.

This work has proved that it is possible to use RCNN based algorithms to detect gesture commands in real time, processing each gesture as a different object to detect by the network. Moreover, it has been proven that, depending on the problem to solve, if it is not very intricate and does not require the detection of a high number of output classes, the use of a smaller CNN offers many advantages over the state-of-the-art published networks, in terms of training time, computation speed, and accuracy. In addition to all this, the use of small CNN networks allows using embedded systems for computation in these type of algorithms. This is an important point when developing this kind of systems.

The results have also been promising. The system detects 96.92% of the gestures correctly, and it is robust to variations that can make other alternative algorithms fail. As an example, the proposed approach is robust to lighting variations and different color skins, provided the training dataset is well created and generalized. This variability makes very difficult to solve skin segmentation approaches for gesture detection. In addition to this, and using a small CNN with a Faster-RCNN architecture, the mean computation time for a 1 Mpix image is 0.14 s using GPU computation, a time that can be taken as real time for systems that use gestures as a form of interaction.

Another point of interest to be taken into consideration and related to the explained results in the above paragraph is that, given the low computational resources that our system needs for real-time gesture detection, it could also be adapted very effectively to other areas beyond the industrial applications, such as domestic devices or smart TVs, for example.

## Figures and Tables

**Table 1:** Main small CNN networks for its use in the RCNN detector.

Network Name	Year of Creation	Depth	Total Number of Layers	Size in Memory	Parameters (Millions)
SqueezeNet	2016	18	68	4.6 MB	1.24
GoogleNet	2015	22	144	27 MB	7.0
Mobilenetv2	2018	53	155	13 MB	3.5

**Table 2:** Hand position for each gesture.

Agree	Halt	Ok	Run
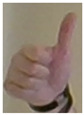	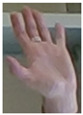	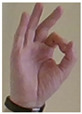	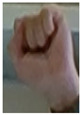

**Table 3:** Detail of the convolutional layers in the Darknet CNN.

Conv. Layer	Filter Size	Num. Filters	Stride	Dilation Factor	Padding
Conv. Layer 1	3 × 3	16	1, 1	1, 1	same
Conv. Layer 2	3 × 3	32	1, 1	1, 1	same
Conv. Layer 3	3 × 3	64	1, 1	1, 1	same
Conv. Layer 4	3 × 3	128	1, 1	1, 1	same
Conv. Layer 5	3 × 3	256	1, 1	1, 1	same
Conv. Layer 6	3 × 3	512	1, 1	1, 1	same
Conv. Layer 7	3 × 3	1024	1, 1	1, 1	same
Conv. Layer 8	1 × 1	1000	1, 1	1, 1	same

**Table 4:** Characteristics of CNN networks used in RCNN detectors.

Network	Year	Layers	Trainable Parameters (Millions)
SqueezeNet	2016	68	1.24
GoogleNet	2015	144	7.0
Mobilenetv2	2018	155	3.5
Darknet	2016	32	8.5
Proposed CNN	2019	24	6.3

**Table 5:** RCNN detector obtained results.

CNN	CCP Metric	Accuracy	Mean Detection Time (s)
Squeezenet	98.33%	99.9%	3.68 s
Googlenet	100%	99.58%	4.57 s
Mobilenetv2	100%	99.99%	8.97 s
Proposed CNN	100%	99.83%	3.54 s

**Table 6:** Fast RCNN detector obtained results.

CNN	CCP Metric	Accuracy	Mean Detection Time (s)
Squeezenet	100%	96.11%	1 s
Googlenet	96.66%	93.65%	1.21 s
Mobilenetv2	98.33%	95. 38%	1.24 s
Proposed CNN	100%	98.64%	0.984 s

**Table 7:** Faster RCNN detector obtained results.

CNN	CCP Metric	Accuracy	Mean Detection Time (s)
Squeezenet	100%	99.83%	0.16 s
Googlenet	100%	99.85%	0.37 s
Mobilenetv2	98.33%	99.86%	0.26 s
Proposed CNN	98.33%	94.19%	0.145 s

**Table 8:** Results of the proposed network architecture using a 10 fold cross validation approach.

Fold	CCP Metric	Mean Accuracy	Mean Detection Time (s)
1	97.56%	99.75%	0.124 s
2	98.45%	97.34%	0.147 s
3	95.3%	95%	0.152 s
4	100%	99.3%	0.161 s
5	96.34%	92.47%	0.153 s
6	98.5%	96.15%	0.139 s
7	94.3%	94.56%	0.17 s
8	97%	99.4%	0.142 s
9	92.8%	97.83%	0.122 s
10	99%	95.8%	0.151 s
Mean	96.92%	96.76%	0.1461 s

## Data Availability

The dataset is private has has been recorded for this work. Due to privacy issues (different persons appearing in the images), it cannot be made open access. Please, contact corresponding author to create an equivalent dataset if needed.
